# *bla*_OXA-181_–positive *Klebsiella pneumoniae*, Singapore

**DOI:** 10.3201/eid1809.111727

**Published:** 2012-09

**Authors:** Tse H. Koh, Delphine Y.H. Cao, Kian S. Chan, Limin Wijaya, Stewart B.G. Low, Mun S. Lam, Eng E. Ooi, Li-Yang Hsu

**Affiliations:** National University of Singapore, Singapore (T.H. Koh, L.-Y. Hsu);; Singapore General Hospital, Singapore (T.H. Koh, D.Y.H. Cao, K.S. Chan, L. Wijaya);; Mount Alvernia Hospital, Singapore (S.B.G. Low);; Mount Elizabeth Medical Centre, Singapore (M.S. Lam);; and Duke–National University of Singapore–Graduate Medical School, Singapore (E.E. Ooi)

**Keywords:** carbapenemase, oxacillinase, bla_OXA-181_, Klebsiella pneumoniae, antimicrobial drugs, antibiotics, drug resistance, bacteria, Bangladesh, Singapore

**To the Editor:** Nordmann et al. (*1*) raised concern over the global spread of carbapenemase-producing *Enterobacteriaceae*. In their article, they called attention to the oxacillinase-48 (OXA-48) type carbapenemases because bacteria that produce these enzymes do not have a distinctive antimicrobial drug susceptibility profile, and there is less awareness of this mechanism of carbapenem resistance. We report the recent isolation of *bla*_OXA-181_–positive *Klebsiella pneumoniae* from 2 patients from Bangladesh who were admitted to separate hospitals in Singapore within a short period of each other.

The first patient was a 64-year-old man who had a recent heart attack and was transferred from a hospital in Dhaka, Bangladesh, to a hospital in Singapore for treatment for pancytopenia. He had no other history of recent travel. While in Bangladesh, the patient had *Pseudomonas* spp. bacteremia and had received meropenem and vancomycin. In Singapore, his antimicrobial drug treatment regimen was changed to ciprofloxacin, linezolid, and amikacin. Blood samples obtained on the day of admission were cultured and grew a vancomycin-resistant *Enterococcus* spp. and a carbapenem-resistant *K. pneumoniae* (isolate DB53879_11).

Two days after admission, when the results of his blood culture were known, the patient’s antimicrobial drug treatment regimen was changed to oral linezolid (600 mg every 12 hours), intravenous tigecycline (initially 50 mg every hour but later increased to 100 mg every 12 hours), and intravenous polymyxin E (initially 3 MU/d but later increased to 3 MU every 12 hours). Blood cultured for *K. pneumoniae* showed positive results for 5 days after the patient was hospitalized before clearing.

Isolate DB53879_11 was resistant to many antimicrobial drugs as determined by Etest (bioMérieux, Marcy l’Etoile, France) (Table). It was strongly positive for carbapenemase production as determined by use of a modified Hodge test (*2*) and showed a negative result with the KPC + MBL Confirm ID Kit (Rosco Diagnostica A/S, Taastrup, Denmark).

**Table:** Antimicrobial drug susceptibilities of 3 *bla*_OXA-181_–positive *Klebsiella pneumoniae* isolates, Singapore*

Drug	MIC (mg/L)		
	DB53879_11	DX1083_11	BL21479_11
Ampicillin	≥256	≥256	≥256
Amoxicillin/clavulanate	≥256	≥256	≥256
Piperacillin/tazobactam	≥256	≥256	≥256
Cefuroxime	≥256	≥256	≥256
Ceftriaxone	≥32	≥32	≥32
Ceftazidime	≥256	≥256	≥256
Cefepime	≥256	≥256	≥256
Ertapenem	≥32	≥32	≥32
Imipenem	16	≥32	16
Meropenem	≥32	≥32	≥32
Doripenem	≥32	≥32	≥32
Ciprofloxacin	≥32	≥32	≥32
Levofloxacin	≥32	≥32	≥32
Gentamicin	≥256	≥256	≥256
Amikacin	≥256	≥256	≥256
Sulfamethoxazole/trimethoprim	≥32	≥32	≥32
Tetracycine	≥256	≥256	8
Tigecycline	4	4	4
Polymyxin B	0.5	1	4

*OXA-181, oxacillinase-181.

Using PCR, we amplified and sequenced a product identical to the complete sequence of *bla*_OXA-181_. Primers designed for known flanking regions of *bla*_OXA-181_ (GenBank accession no. JN205800) failed to amplify any product. Like described isolates (*3*–*5*), DB53879_11 was also positive for *bla*_OXA-1_ and *bla*_CTX-M-15_, but it also was positive for *bla*_CMY-4_. An attempt to transfer *bla*_OXA-181_ to azide-resistant *Escherichia coli* J53 by plate mating was unsuccessful.

Two weeks after we received the specimen from the first patient, we were referred 2 carbapenem-resistant *K. pneumoniae* strains isolated from sputum (isolate DX1083_11) and blood (isolate BL21479_11) from a 30 year-old man admitted to another hospital in Singapore. He had been treated in the same hospital in Dhaka as the first patient for multiorgan failure secondary to dengue shock syndrome. Antimicrobial drug susceptibility phenotypes and resistance gene complements for DX1083_11 and BL21479_11were similar to those for the isolate from the first patient. The second patient received intravenous tigecycline, polymyxin B, and meropenem.

All 3 isolates were identical when tested by random amplification of polymorphic DNA (*6*) and by pulsed-field gel electrophoresis after restriction endonuclease digestion of chromosomal DNA with *Spe*I. Multilocus sequence typing showed that DX1083_11 belonged to sequence type 14 (www.pasteur.fr/recherche/genopole/PF8/mlst/Kpneumoniae.html). This sequence type is the same as that for *bla*_OXA-181_–positive *K. pneumoniae* reported from New Zealand (*3*). Both patients died of their illnesses.

OXA-181 is a close relative of OXA-48 from which it differs by 4 aa (*4*). *bla*_OXA-181_–positive *K. pneumonia* infections were first described in India but imported cases have since been described in Oman, the Netherlands, and New Zealand (*3*–*5*,*7*). We were unable to confirm the original source of these isolates, and continuous surveillance for carbapenemase producers in our hospital has not uncovered any *bla*_OXA-181_–positive isolates since 1996. To our knowledge, there are no reports of *bla*_OXA-181_–positive isolates in Bangladesh. However, this country borders India, which is a source of *bla*_OXA-181_–positive *Enterobacteriaceae*. These cases highlight potential problems that may arise from medical tourism (the rapidly increasing practice of traveling across international borders to obtain health care) and document the expanding range of a newly emerging mechanism of carbapenem resistance.
